# A Novel Vehicle Stationary Detection Utilizing Map Matching and IMU Sensors

**DOI:** 10.1155/2014/597180

**Published:** 2014-09-07

**Authors:** Md. Syedul Amin, Mamun Bin Ibne Reaz, Salwa Sheikh Nasir, Mohammad Arif Sobhan Bhuiyan, Mohd. Alauddin Mohd. Ali

**Affiliations:** Department of Electrical, Electronic and Systems Engineering, Universiti Kebangsaan Malaysia (UKM), 43600 Bangi, Selangor, Malaysia

## Abstract

Precise navigation is a vital need for many modern vehicular applications. The global positioning system (GPS) cannot provide continuous navigation information in urban areas. The widely used inertial navigation system (INS) can provide full vehicle state at high rates. However, the accuracy diverges quickly in low cost microelectromechanical systems (MEMS) based INS due to bias, drift, noise, and other errors. These errors can be corrected in a stationary state. But detecting stationary state is a challenging task. A novel stationary state detection technique from the variation of acceleration, heading, and pitch and roll of an attitude heading reference system (AHRS) built from the inertial measurement unit (IMU) sensors is proposed. Besides, the map matching (MM) algorithm detects the intersections where the vehicle is likely to stop. Combining these two results, the stationary state is detected with a smaller timing window of 3 s. A longer timing window of 5 s is used when the stationary state is detected only from the AHRS. The experimental results show that the stationary state is correctly identified and the position error is reduced to 90% and outperforms previously reported work. The proposed algorithm would help to reduce INS errors and enhance the performance of the navigation system.

## 1. Introduction

Navigation has been used since ancient days. Over the time, the sources of the navigation systems have evolved from the sun and stars to the modern day navigation sensors [1–3]. Modern navigation has been utilizing the various sensors to locate the position of a vehicle for fleet management, location based service, asset tracking, intelligent vehicle control, and so forth [4]. GPS and INS are popular navigation devices of the modern days. GPS can provide excellent navigation in low update rate with a good accuracy where the satellite signals are not obstructed [5]. However, the performance severely degrades or even complete outage occurs in tunnels, in urban areas with skyscrapers, or in parking garages. On the other hand, the INS can provide continuous navigation information with high data rates which does not depend on any external sources like the satellites of the GPS [6]. But the downside is that the accuracy of the INS largely depends on the quality of the IMU sensors.

IMU conventionally consists of three orthogonal accelerometers and gyroscopes. Besides, the magnetometer is also widely used nowadays. Theoretically, single and double integration of the rotation rate of gyro and acceleration from the accelerometer outputs provide velocity and position information. But in reality the nonlinearity, bias, drift, and noise of the sensors make accuracy of the predicted trajectory valid for short time only. The recent advances in MEMS lead to very inexpensive IMU sensors [7]. But the bias, drift, noise characteristics, and overall accuracy are inferior [8]. The gyroscope of a low cost IMU cannot sense the rotation of the earth like the gyroscope of a tactical grade IMU [9]. Moreover, the acceleration sensed from the accelerometer of the low cost IMU is very noisy besides the bias error [10]. Thus, the position derived from the double integration of the acceleration sensed from the accelerometer and the orientation derived from the accelerometer and gyroscope give rise to an error in position which increases as the cube of time due to drift, bias, and other noises [11].

The performance of a low cost MEMS based INS can be improved significantly. The biases and the drifts of the sensors can be improved by integrating with an aiding measurement like GPS [12]. However, the performance of the GPS degrades or even a complete outage can occur due to the line of sight problem which in turn affects the navigation performance of the low cost INS. This inspires the researchers for alternative techniques to reduce the bias and drift errors of the low cost IMU. Several researchers suggested the stationary update as an alternative technique which is popularly known as zero velocity update (ZUPT). 

The ZUPT is widely used in pedestrian navigation system [13]. For a vehicle navigation system, the ZUPT mode utilizes the zero velocity condition or the stationary state to estimate the systematic errors of the IMU sensors [14]. When stationary, the only forces acting on the unit are the gravitational force and the rotation of the earth. Beyond these forces, others are the errors in the form of bias, noise, or drift which can assist the popular estimation filters like Kalman filter in getting the differences between the static model and the actual observation to estimate. In urban areas, these stationary states occur frequently due to the traffic congestion and traffic signals in intersections. Thus, the correction can be performed at regular interval and the estimation error of the low cost INS can be kept minimum during a GPS outage [14]. However, the decision of false stationary mode is more severe than of a false moving as the force caused by the movement can be detected as the sensor noise and thereby affects the position accuracy negatively [15]. Thus, the detection of a correct stationary state is crucial. 

Several researchers worked on various methods for detecting the ZUPT or the stationary state. The majority of the works were focused on pedestrian and indoor navigation where the sensor errors were corrected when the foot of the pedestrian touched the ground [13]. These approaches are not applicable to land vehicle systems [15]. The controlled conditions were imposed to limit vibration and ensure immobility in [16–19] to calibrate and align high performance systems. However, these constraints cannot be imposed in land vehicle applications such as intelligent transportation systems [15]. Grejner-Brzezinska et al. proposed a stationary update system for land vehicles where the user is alerted to perform stationary update manually when the escalation of position error covariance crosses a certain threshold during a GPS outage [14]. Due to the manual stationary update, this system is not also practical for land vehicle application. The GPS velocity threshold of >20 s was proposed by [20] to detect a stationary state which would not function in a GPS degraded condition. Moreover, with the GPS availability, the stationary measurement is not necessary. In [21], the stationary sate was detected only from the accelerometer data with a lengthy time window of 15 s. This long time window may miss stationary states as vehicle may not be stationary for such a lengthy period. A pattern recognition approach was proposed by [22] to detect stationary state using the cumulative accelerations only from the accelerometers. But the algorithm cannot detect a reliable stationary state when the moving speed is very low as mentioned by the author [22]. Ramanandan et al. [15] proposed a frequency domain approach using only accelerometers and gyroscopes which can suffer from false detection in low acceleration. But none of the researchers utilized the acceleration, heading, and roll and pitch information along with the MM intersection identification for a confirmed stationary state detection.

The trajectory and location of a vehicle are restricted by the road network. Thus, the digital road network map can be used to impose these constraints on the navigation solution for a vehicle. MM algorithms integrate data from one or more positioning sensors with the spatial road network data from a set of candidate links to identify the correct link on which a vehicle is travelling and determine the physical location of the vehicle on the link [23]. The main focus of MM algorithms is to reduce the positioning error by matching the travelled vehicle route to the best fitted set of road segments of a digital road map. Besides, MM can also correctly identify the intersection of the road. A vehicle needs to stop at the intersections frequently. By using the intersection information, a vehicle can be more correctly identified for its stationary state.

This paper proposes a novel stationary detection technique by the threshold approach of acceleration, heading, and pitch and roll of an AHRS built from the IMU sensors and combining with the correct identification of road intersection. An improved MM algorithm with multiple road junctions based on the matching weight values is used to identify the road intersection where vehicle is likely to stop. The rest of the paper is organized as follows: the Sensor Model and AHRS section introduces the sensor models and the fusion of IMU sensors for AHRS; MM for Detecting Intersections section describes the technique of detecting traffic signal from digital map and GPS/INS; ZUPT Theory and Detection of Stationary State section describes the proposed algorithm of stationary detection from AHRS and MM information; Experimental Results section presents the hardware setup and the result of the test for stationary detection followed by the performance analysis where the navigational performance with stationary update is presented and compared. 

## 2. Sensor Model and AHRS 

MEMS based IMUs are generally equipped with triaxial accelerometers and gyroscopes. Some of the systems have triaxial magnetometers also. These sensors are aligned with the body (b) frame where *x*-axis is along the forward movement direction of the vehicle, *y*-axis is towards the right side of the vehicle, and *z*-axis follows the orthogonal right handed rule [24]. Body frame axes are also aligned with roll, pitch, and yaw axes. A generalized inertial sensor model is given in (1) [15]. Consider

(1) $$\begin{array}{c} {\tilde{y}}_{b}=y_{b}+b_{b}+n, \end{array}$$

where ${y_{b}}^{T}=\left[\begin{array}{cc} {f_{b}}^{T} & {\omega_{ib}^{b}}^{T} \end{array}\right]$ and *f*_*b*_ = *a*_*b*_ + _*n*_^*b*^ *Rg*_*n*_ is the specific force vector. The vector *y*_*b*_ is corrupted by measurement noise *n* and slowly varying bias ${b_{b}}^{T}=\left[\begin{array}{cc} {}_{\;}^{a}{b_{b}}^{T} & {\;}_{\;}^{g}{b_{b}}^{T} \end{array}\right]$, where _  _^*a*^ *b*_*b*_ and _  _^*g*^ *b*_*b*_ are the accelerometer and gyroscope biases. *a*_*b*_ denotes the acceleration in body frame and *g*_*n*_ is the gravity vector in navigation frame expressed as ${g_{n}}^{T}=\begin{array}{ccc} 0 & 0 & g_{e} \end{array}$, where *g*_*e*_ ≈ −9.8 m/s^2^. All the biases are modelled as random walk.

The INS error state vector can be expressed by (2). Consider

(2) $$\begin{array}{c} \delta x^{T}=\left[\begin{array}{ccccc} {}_{\;}^{b}\delta {p_{n}}^{T} & {}_{\;}^{b}\delta {v_{n}}^{T} & {\rho_{n}}^{T} & {}_{\;}^{a}\delta {b_{b}}^{T} & {}_{\;}^{g}\delta {b_{b}}^{T} \end{array}\right], \end{array}$$

where ^*b*^ *δp*_*n*_ denotes the position error and ^*b*^ *δv*_*n*_ denotes the velocity error in navigation frame. ^*a*^ *δb*_*b*_ and ^*g*^ *δb*_*b*_ denote the accelerometer and gyroscope bias in body frame, respectively. *ρ*_*n*_^*T*^ denotes the small rotation angle transformed from estimated to actual navigation frame.

The differential equations for the errors states are given in (3). Consider

(3) bδpn˙=bδvn,bδv˙n=−[fn×]ρn+bnRaδbb+ bnRan,ρ˙n=bnRgδbb+bnRgn,gδb˙b=gnb,aδb˙b=anb,

where ^*a*^ *b* and ^*g*^ *b* are the measurement noise of the accelerometer and gyroscope.

By utilizing the IMU sensors, an AHRS can be constructed which can provide the orientation of a vehicle in terms of yaw and pitch and roll information. The integrated output of the gyroscope combined with accelerometer output can pin down the drift of the gyroscope and can provide roll and pitch information. But the accelerometer cannot provide any yaw information. The integrated output provided by the gyroscope alone cannot be trusted as the low cost gyroscope drifts quickly. The magnetometer can aid to get the better yaw or heading information with its reading in relation to Earth's magnetic field. Thus, a fusion algorithm is required to determine the roll, pitch, and yaw information. 

The Euler angles are adopted for orientation representation in the fusion algorithm. The second-order complementary filter is used to determine the roll and pitch from the accelerometer and gyroscope due to its simplicity and ease of implementation [25]. The roll and pitch of a vehicle can be found from the vehicle's gravity vector sensed from the accelerometer. The equation of motion of a vehicle in terms of specific force from a three-axis accelerometer in body frame can be expressed by (4) [26]. Consider

(4) [axayaz]=[u˙v˙w˙]+[0w−v−w0uv−u0][gxgygz]+[−gz2−gy2gxgy−gz2gxgz+g˙ygxgy+g˙z−gx2−gz2gzgy−g˙xgxgz−g˙ygzgy+g˙x−gy2−gx2][lxlylz]+g[sinθ−cos⁡θsinϕ−cos⁡θcos⁡ϕ],

where *a*_*x*_, *a*_*y*_, and *a*_*z*_ are forces from the three-axis accelerometers; *u*, *v*, and *w* are the linear velocity components along the three axes in the body frame with the origin at the center of gravity; *l*_*x*_, *l*_*y*_, and *l*_*z*_ are the accelerometers coordinates along each axis in the same body frame with the origin at the center of gravity; *g*_*x*_, *g*_*y*_, and *g*_*z*_ are the three-axis gyroscope angular rate; *ϕ* and *θ* are the roll and pitch, respectively. As it is difficult to measure *v* and *w* and their derivatives, the true attitude cannot be measured from (5). So, the simplified equations for roll (*ϕ*_*a*_) and pitch (*θ*_*a*_) determination for accelerometers are given in (6). Consider

(5) $$\begin{array}{c} \phi_{a}=\tan^{-1}\left(\frac{a_{y}}{a_{z}}\right), \end{array}$$

(6) $$\begin{array}{c} \theta_{a}=\tan^{-1}\left(\frac{a_{y}}{\sqrt{{a_{y}}^{2}+{a_{z}}^{2}}}\right). \end{array}$$

The roll, pitch, and yaw of a vehicle can be measured from the three-axis gyroscopes in the body frame. The equation for determining the roll, pitch, and yaw from the gyroscopes through the Euler Kinematics is given in (7) [26]. Consider

(7) $$\begin{array}{c} \left[\begin{array}{c} {\dot{\phi }}_{g}\\ {\dot{\theta }}_{g}\\ {\dot{\psi }}_{g} \end{array}\right]=\left[\begin{array}{ccc} 1 & \sin\phi \tan\theta & \cos\phi \tan\theta \\ 0 & \cos\phi & -\sin\phi \\ 0 & \sin\phi \text{sec}\;\theta & \cos\phi \text{sec}\;\theta \end{array}\right]\left[\begin{array}{c} g_{x}\\ g_{y}\\ g_{z} \end{array}\right]. \end{array}$$

Magnetometer is the digital version of a conventional compass that can show the magnetic north direction. Thus, a magnetometer can be used to find out the heading of a vehicle. The equation for the yaw angle determination from the measured magnetometer strength in body frame is expressed in (8). Consider

(8) $$\begin{array}{c} \psi_{m}=\tan^{-1}\left(\frac{m_{z}\sin\phi -m_{x}\cos\phi }{m_{x}\cos\theta +\sin\theta \left(m_{y}\sin\phi +m_{z}\cos\phi \right)}\right), \end{array}$$

where *m*_*x*_, *m*_*y*_, and *m*_*z*_ denote the magnetic field strength component expressed in the body frame.

**Figure 1:**
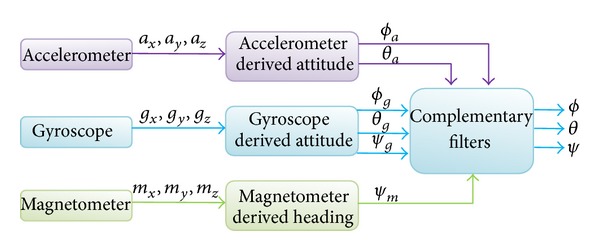
Block diagram of the fusion algorithm for AHRS.

The fusion algorithm utilizes the complementary filter where the gyroscope derived attitude is passed through a high-pass filter and the accelerometer derived attitude is passed through the low pass filter. The block diagram of the fusion algorithm is shown in Figure 1. The fusion of the roll angles from gyroscope and accelerometer is done in a complementary filter. Another complementary filter is used for the fusion of pitch angles derived from the gyroscope and accelerometer. The heading derived from the gyroscope is corrected with the heading derived from the magnetometer and the fusion is done by another complementary filter.

**Figure 2:**
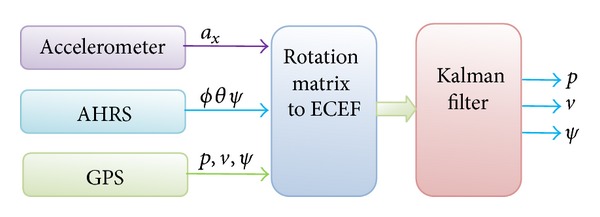
Block diagram of GPS/INS for position, velocity, and heading.

A Kalman filter is used for the purpose of fusion to determine the position *p*, velocity *u*, and heading Ψ from the GPS, AHRS, and accelerometer following [27] as shown in Figure 2. The information from the accelerometer in the longitudinal axes and the heading information from the AHRS are converted to the earth centered earth fixed (ECEF) frame following [24]. GPS receiver outputs data defined by the specification of the National Marine Electronics Association (NMEA). GPRMC is of one of the NEMA format that contains position, velocity and heading information in ECEF frame [28]. The gravity vector is deducted from the accelerometer caused by any tilt. Then, the accelerometer components are added to determine the velocity. This is again added to determine the position. However, this position contains large errors due to noise, bias, and drifts which will be corrected by detecting the stationary update later. The position error is corrected whenever the GPS information is available.

## 3. MM for Detecting Intersections

**Figure 3:**
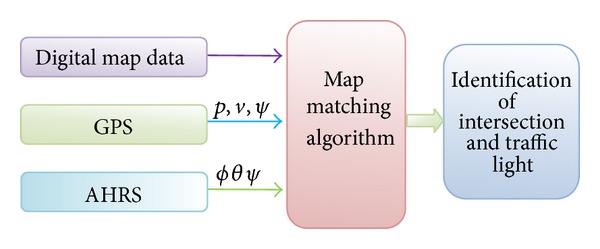
Sensor integration for MM.

Map matching algorithms integrate positioning data with spatial road network data to identify the correct link on which a vehicle is travelling and to determine the correct location of a vehicle on the road [29]. The positioning data is integrated from GPS, AHRS, and digital map data in a complementary manner as shown in Figure 3. An improved MM algorithm with multiple road junctions based on the matching weight values is used to identify the road intersection where vehicle is likely to stop in traffic signal. An initial analysis procedure with two sampling points before entering the intersections and after exiting the intersections is searched. The search results are combined with entrance link and given a weight after being conjoined. These data are used in the temporal analysis together with the speed data to choose the best route. Thus, the matching errors are reduced and the accuracy is increased which can identify the intersections correctly.

**Figure 4:**
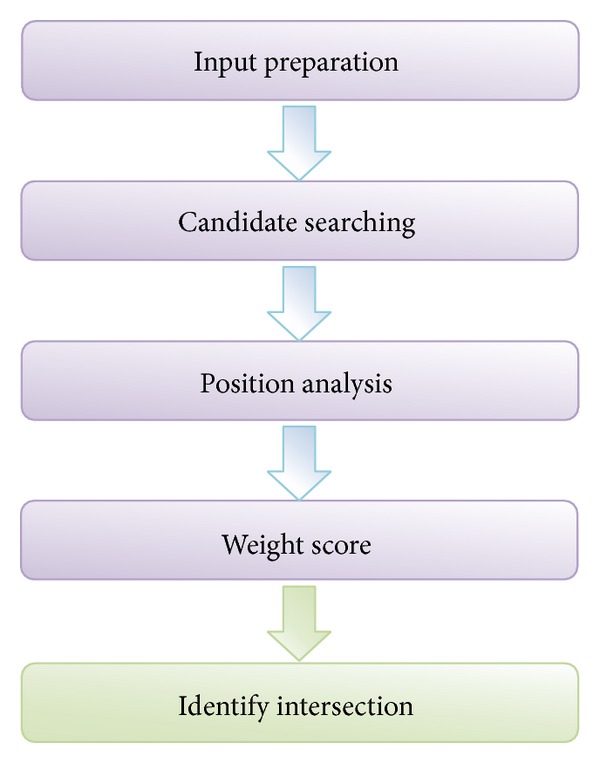
Steps of the proposed MM algorithm to identify the intersection.

The MM algorithm proposed in this paper is composed of 5 major modules: input preparation, candidate searching, position analysis, weight score, and identify intersection. The first step prepares the input data from GPS/INS and digital road network database. Second step is the beginning of the MM process, which finds the candidate point (CP) around the GPS/INS point. The third step is the position analysis step where it performs initial analysis, spatial analysis, and temporary analysis. The fourth step is the measurement of the value of the weight. The final step updates the matching result on the nearest road segment based on the weight from the fourth step. The steps of the proposed algorithm are shown in Figure 4.

### 3.1. Input Preparation

**Figure 5:**
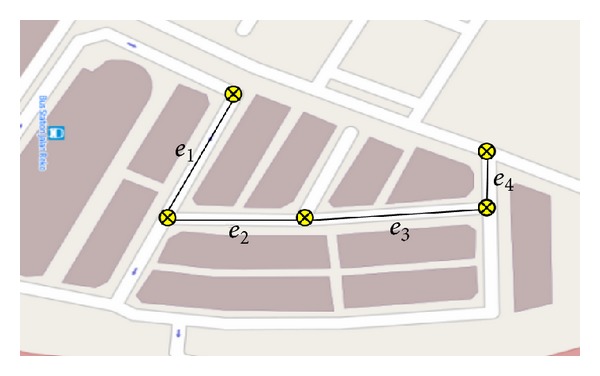
Start and end point of road segments.

In the input preparation step, the data from the GPS/INS and digital road network database are processed for the subsequent steps. The latitude, longitude, and timestamp for each data point are taken. The sequence of the points are known as trajectories where data points are *T* = {*P*_1_ → *P*_2_ → *P*_3_ → ⋯*P*_*n*_}. The value of *T* for each point includes a set of candidate road segments (CRS) with radius *r*. The CRS contains entire road segments that are associated with line segment. The line segment information contains starting point, ending point, and typical travel speed in the map database. The OpenStreetMap (OSM) of Quantum Geographical Information System (QGIS) has been used as the map database. QGIS provides data in shapefile format comprised of latitude and longitude, roadway type, and speed limitation. The start and end of road segments are given in coordinate form as shown in Figure 5 for an intersecting road.

### 3.2. Candidate Searching

**Figure 6:**
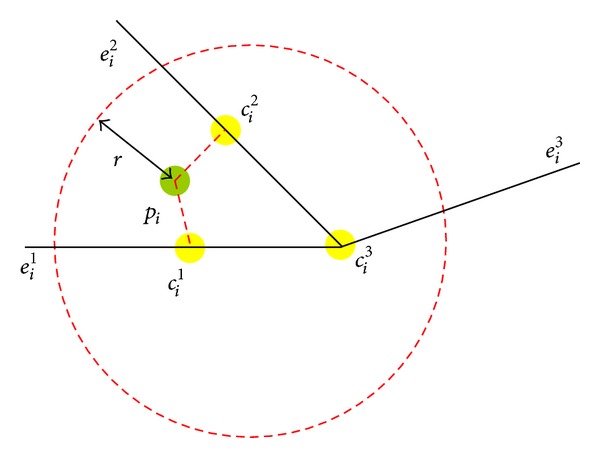
Illustration of CRS and CP.

The second step of MM algorithm is the candidate searching. Candidate searching contains the road network database with indexed edge and vertex information. The sampling point projection onto *e* between its endpoints is selected as a CP. Projection of geometry is chosen when the endpoints are close to sampling points considering Euclidean distance equation.  Figure 6 illustrates CP_1_ = {*c*_1_^1^, *c*_1_^2^, *c*_1_^3^} and CRS_1_ = {*e*_1_^1^, *e*_1_^2^, *e*_1_^3^} for each *P*_*i*_ with radius *r*.

### 3.3. Position Analysis

**Figure 7:**
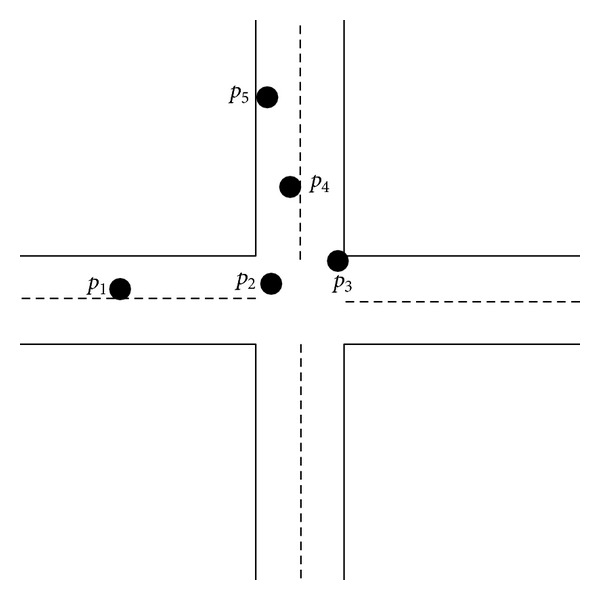
Position errors of GPS/INS points at intersection.

**Figure 8:**
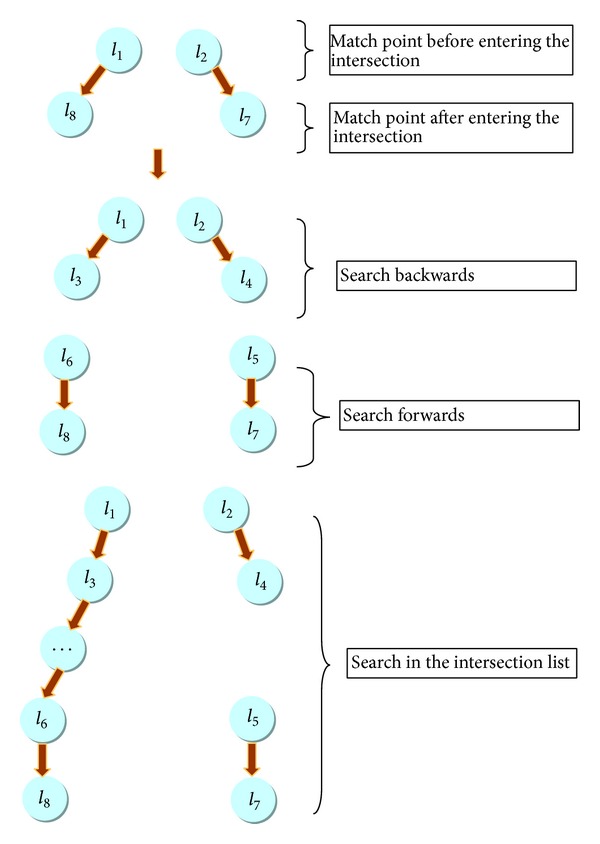
Search process of the initial analysis.

The third step of MM algorithm is the position analysis, which reduces the map projection errors and navigation sensor errors. The projection errors and the GPS/INS positional errors are shown in Figure 7. This module consisted of initial match of sampling point, spatial and temporary analysis. The position analysis is the initial match of sampling point. A search method is used to find the road link from the road network as shown in Figure 8. A vehicle traveling through the intersection contains 4 sampling points. The first point is matched to the links *l*_1_ and *l*_2_. When the second and third points are in intersection, the searching point continues until the fourth point. It matches the links *l*_7_ and *l*_8_. This process continues searching subsequent links of *l*_1_ and *l*_2_ until it reaches the entrance links *l*_3_ and *l*_4_ of the intersection. Then, it continues searching the previous links of *l*_7_ and *l*_8_ until reaching the exit links *l*_5_ and *l*_6_ of the intersection. The intersection list will be found by searching the route between *l*_3_, *l*_4_ and *l*_5_, *l*_6_. Lastly, the traveling route can figure out the matching link.

Based on the result of the initial analysis, the process of MM continues for a second analysis called spatial analysis. The main purpose of spatial analysis is to construct candidate graph *G*′(*V*′, *E*′). The error of GPS/INS measurement can be described as normal (Gaussian) distributions *N*(*μ*, *σ* ^2^). For a CP *c*_*i*_^*j*^, its observation probability with respect to *p*_*i*_ is given in (9). The observation probability does not consider sudden switch and a previous match point but only the distance between CP and GPS/INS points. The transition probabilities are introduced to solve this problem. Consider

(9) $$\begin{array}{c} N\left(c_{i}^{j}\right)=\frac{1}{\sqrt{2\pi \sigma }}e^{{\left(x_{i}^{j}-\mu \right)}^{2}/2\sigma^{2}}, \end{array}$$

where *x*_*i*_^*j*^ is the Euclidean distance between *c*_*i*_^*j*^ and *p*_*i*_, *σ* is the standard deviation, *σ* ^2^ is the corresponding variance, and *μ* is the mean (expectation).

After computing the value of probability, the spatial analysis can be defined as (10). These spatial analyses represented measuring the similarity of the vehicle movement from the position *c*_*i*−1_^*t*^ to *c*_*i*_^*s*^ and computed any pair of two neighbouring CPs. Consider

(10) $$\begin{array}{c} F_{s}\left(c_{i-1}^{t}-c_{i}^{s}\right)=N\left(c_{i}^{s}\right)\times V\left(c_{i-1}^{t}⟶c_{i}^{s}\right),\;2\leq i\leq n. \end{array}$$

Based on the result of the spatial analysis, the process of MM continues for a third analysis called temporary analysis. Temporary analysis determines the correct link that has been in spatial analysis without considering the value of speed constraints of the *e*. To measure the speed information between the two paths, the exact average speed of the vehicle has to be determined. The average speeds of the vehicle on *c*_*i*−1_^*t*^ and *c*_*i*_^*s*^ can be determined by (11). Consider

(11) Ft(ci−1t⟶cis) =∑u=1k(eu′·v×v¯(i−1,t)→(i,s))∑u=1k(e′·v)2×∑u=1kv2(i−1,t)→(i,s),

where *e*_1_′ → *e*_2_′ ⋯ →*e*_*k*_′ is the shortest path between *c*_*i*−1_^*t*^ and *c*_*i*_^*s*^ and ${\bar{v}}_{\left(i-1,t)\to (i,s\right)}$ is the average speed from *c*_*i*−1_^*t*^ to *c*_*i*_^*s*^ which can be simply computed by (12). Consider

(12) $$\begin{array}{c} F\left(c_{i-1}^{t}⟶c_{i}^{s}\right)=F_{s}\left(c_{i-1}^{t}⟶c_{i}^{s}\right)\times F_{t}\left(c_{i-1}^{t}⟶c_{i}^{s}\right). \end{array}$$

**Figure 9:**
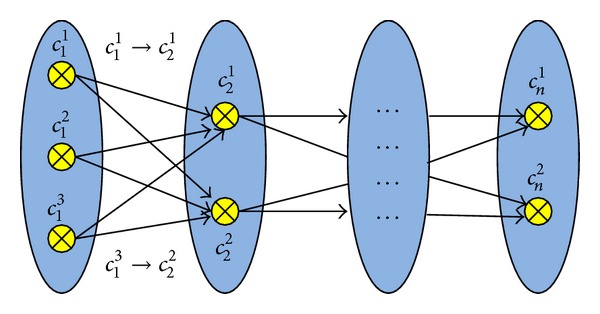
Candidate graph.

After spatial and temporal analysis, candidate graphs are built as illustrated in Figure 9. The graph nodes are set of CPs for GPS/INS points. The graphs edges are set of shortest paths between two adjacent CPs. These nodes and edges are weighted with the values on the result of the temporal and spatial analysis.

### 3.4. Weight Score

The weight scores are built as *M* = diag⁡{*M* ^(1)^, *M* ^(2)^,…, *M* ^(*n*)^} based on the generated candidate graph in the previous module, where *M* ^*i*^ = (*m*_*ts*_^(*i*)^)_*a*_*i*−1_×*a*_*i*__ = (*F*(*c*_*i*−1_^*t*^→*c*_*i*_^*s*^))_*a*_*i*−1_×*a*_*i*__. The weighted influence of the CP defines *a*(*n* − 1) dimension distance weight *W*_*i*_ for every sampling point *p*_*i*_. For each *p*_*i*_, the matrix of weight represents the similarity of all *e* with the actual path as remote influence. The diagonal matrix gives the value of weights for all other points to *p*_*i*_ combined with the distance to *p*_*j*_. Equation (13) is computed with *i* = 2,3,…, *n* for *W*_*i*_. The values *w*_*i*_^(*j*)^ = *f*(^dist⁡(*p*_*i*_,*p*_*j*_)^), *j* = 1,2,…, *n*, and dist⁡(*p*_*i*_, *p*_*j*_) are the Euclidean distance between *p*_*i*_ and *p*_*j*_. The weight score is constructed where the distance weight shows the minimum value. This minimum value is used in the next module to find the best matching result. Consider

(13) $$\begin{array}{c} w_{i}=\mathrm{diag}\left\{w_{i}^{\left(2\right)},w_{i}^{\left(2\right)},\ldots ,w_{i}^{\left(i+1\right)}\right\},\\ w_{1}=\left\{w_{i}^{\left(2\right)},w_{i}^{3},\ldots ,w_{i}^{\left(n\right)}\right\}. \end{array}$$

### 3.5. Identify Intersection

The identify intersection is the final step. This module updates the matching result on the nearest road segment according to result of weighted score. In the position analysis step, a candidate graph *G*_*T*_′(*G*_*T*_′, *E*_*T*_′) is generated for trajectory *T* = *p*_1_ → *p*_2_ → ⋯*p*_*n*_. *V*_*T*_′ is a set of CPs for every GPS/INS sampling point, and *E*_*T*_′ is a set of edges representing the shortest path between any two neighbouring CPs as depicted in Figure 9. Each node in *G*′ is associated with *N*(*c*_*i*_^*j*^). Each edge is associated with *V*(*c*_*i*−1_^*t*^ → *c*_*i*_^*s*^) and *F*_*t*_(*c*_*i*−1_^*t*^ → *c*_*i*_^*s*^).  Table 1 shows an example of matched sequence procedure. The initial observation algorithm fills out Table 2 for *N*. The matching result for each CP is calculated. 

Candidate *c*_2_^1^ is considered first. The paths *c*_1_^1^ → *c*_2_^1^ are evaluated as *F*(*c*_1_^1^ → *c*_2_^1^) = 0.3∗0.5 = 0.15. Similarly *F*(*c*_1_^2^ → *c*_2_^1^) = 0.3∗0.3 = 0.09 and *F*(*c*_1_^3^ → *c*_2_^1^) = 0.3∗0.4 = 0.12. Therefore, *f*[*c*_2_^1^] = max⁡(0.8 + 0.15), (0.5 + 0.09), (0.3 + 0.12) = 0.95 and *c*_2_^1^'s parent is *c*_1_^1^. The process is repeated for all the candidates.

The highest overall score is *c*_3_^2^ and is selected as the matching result for the last GPS/INS point. Finally, the matching intersection result is *c*_1_^1^, *c*_2_^2^, and *c*_3_^2^. Thus, the vehicle can be correctly identified on the road. With the correct positioning of the vehicle on the road, the correct intersection and the traffic light can be identified where the vehicle is likely to stop.

## 4. ZUPT Theory and Detection of Stationary State

The stationary condition of a vehicle allows improving the navigational performance of a low cost IMU. When the vehicle is in rest position, the only forces acting on are the gravity of the earth and the force from the earth rotation [30]. So, when the vehicle is in stationary position, the velocity (*v*_*b*_) and angular rate (*ω*_*b*_) should be zero. The difference between the static and actual observation can be used in the Kalman filter to estimate the errors. With the imposed zero measurement velocity, the measurement and the linearized model of the velocity residuals are calculated by (14) and (15), respectively. The measurement and the linearized model of the angular rate residuals for the gyroscope are calculated by (16) and (17), respectively. Consider

(14) $$\begin{array}{c} \delta {v_{b}}^{n}={{\tilde{v}}_{b}}^{n}-{{\hat{v}}_{b}}^{n}=-{{\hat{v}}_{b}}^{n}, \end{array}$$

(15) $$\begin{array}{c} \delta {v_{b}}^{n}=H_{v}\delta x, \end{array}$$

(16) $$\begin{array}{c} \delta {\omega_{b}}^{n}={{\tilde{\omega }}_{b}}^{n}-{{\hat{\omega }}_{b}}^{n}=-{{\hat{b}}_{b}}^{n}, \end{array}$$

(17) $$\begin{array}{c} \delta {\omega_{b}}^{n}=H_{\omega }\delta x. \end{array}$$

The stationary detection algorithm needs to be designed keeping the easy implementation, simplicity, practicality, and robustness in consideration. The premise of the proposed approach is that IMU reading for vehicle in motion or at constant cruise will have inconsistent reading due to road condition and engine vibration. This can be utilized to detect a stationary condition. Thus, a reliable stop detection algorithm can be one that is based on the analysis of the variance of an acceleration, heading, roll, and pitch. The accelerometer output during the movement of a vehicle varies significantly due to the engine vibration, road condition, wind, and so forth, compared with the stationary states. In a stationary state, the accelerometer output includes only gravity, bias, and noise accompanied with the environmental disturbances. The heading from the AHRS output built from IMU also varies significantly, while the vehicle is in moving condition compared with a stationary condition [31]. Similarly, the roll and pitch of the vehicle also varies in moving condition compared with the stationary condition. The stationary detection from the acceleration, heading, and roll and pitch can be combined with the identified intersection or traffic light from the map matched algorithm to confirm a stationary state.

A land vehicle generally plies on flat roads where ramps are generally less than 5° [32]. Thus, the vertical or the *z*-axis accelerometer shows the local gravity added with the road vibration. As such, the output of the *z*-axis accelerometer does not vary significantly in a stop or moving condition [32]. Similarly, the output of the *y*-axis accelerometer also does not vary significantly. But the *x*-axis accelerometer along the longitudinal axis varies significantly as the vehicle accelerates or decelerates during the movement. In a complete stop, the variance of the *x*-axis accelerometer contains only the signals due to the engine vibration in addition to the bias and noise. Thus, the *x*-axis accelerometer output plays an important role in detecting the stationary state. The variance of the *x*-axis accelerometer $||{\vec{a}}_{x}||$ does not cross a certain threshold in a stationary state. This can be found empirically with the data inside the time window of a predetermined length. The mean *μ*_*a*_ and the variance *σ* ^2^_*a*_ of the accelerometer are computed. The mean quadratic deviation of the acceleration samples from *μ*_*s*_ time window is calculated by (18), where *N* is the number of samples inside the time window. The stationary state is detected based on the deviation *σ*_*n*_ and variance *σ*_*a*_ of the *x*-axis accelerometer. When *σ*_*n*_ < *c*_*a*_ · *σ*_*a*_, the vehicle is considered stationary. The value of the *c*_*a*_ is selected empirically. Consider

(18) $$\begin{array}{c} \sigma_{n}=\sqrt{\frac{1}{N}\overset{N}{\underset{k=1}{\sum }}{\left(||{\vec{a}}_{k}||-\mu_{a}\right)}^{2}}. \end{array}$$

When the vehicle is in moving condition, the heading of the vehicle changes. Even in a straight road, the heading also changes slightly. The little change in the heading occurs due to the road condition and the engine vibration. But in a stationary condition, the change in heading is trivial. Thus, heading change can be distinguished from the moving condition. The mean *μ*_*ψ*_ and the variance *σ* ^2^_*ψ*_ of the heading are computed. The mean quadratic deviation of the heading from *μ*_*s*_ time window is calculated by (18), where *N* is the number of samples inside the time window. The stationary state is detected based on the computed deviation *σ*_*n*_ and variance *σ*_*ψ*_ of the heading and the fulfilment of the condition *σ*_*n*_ < *c*_*ψ*_ · *σ*_*ψ*_. The value of the *c*_*ψ*_ is selected from test drive.

The roll and pitch of a land vehicle does not vary like an air vehicle. Still, the roll and pitch of a land vehicle varies in a moving condition due to road imperfection and engine vibration. But in a stationary condition, the roll and pitch of the vehicle changes only due to the engine vibration. This change is distinguishable from the moving condition which can be found from the computed deviation *σ*_*n*_ and the variance *σ*_*ϕ*_ of the roll and variance *σ*_*θ*_ of the pitch. The conditions *σ*_*n*_ < *c*_*ϕ*_ · *σ*_*ϕ*_ and *σ*_*n*_ < *c*_*θ*_ · *σ*_*θ*_ are used for roll and pitch, respectively, where the *c*_*ϕ*_ and *c*_*θ*_ are selected empirically.

The false stationary detection is catastrophic as it can corrupt the bias and thereby the navigational information. As such, the proposed methodology emphasizes the correct stationary detection. A longer time window of 15 seconds as proposed in [21] may miss the stationary states. This motivates the selection of a suitable time window sufficient to detect a correct stationary state. When the MM algorithm identifies the intersection and at the same time the AHRS based detection also identifies the stationary state, the probability of false detection is nil. But when the stationary state is only detected from the AHRS, the possibilities of false stationary state increase. This motivates the selection of a variable time window. Thus, when the MM algorithm identifies the intersection in conjunction with AHRS based stationary detection, a smaller time window of 3 s is applied. A longer time window of 5 s is applied when the stationary detection is only based on the acceleration, heading, roll, and pitch. The premise for a shorter time window for the MM position is that the vehicle is likely to stop at the intersection. When the traffic signal or the intersection is detected, the acceleration, heading, and pitch and roll are also checked for the stationary detection. The acceleration, heading, and roll and pitch in addition to the intersection eliminate the possibilities of false detection. Besides the intersection, a vehicle may stop due to traffic congestion or for any other reasons. During such a stop, a longer time window is applied for detecting the stationary state from the acceleration, heading, and pitch and roll. This longer time window can ensure a confirmed stationary state. Thus, the combination of the variable time window, MM intersection detection and acceleration, heading, and pitch and roll based detection eliminate the possibilities of false stationary detection.

## 5. Experimental Results

The experiment utilized a cheap HI-204III GPS receiver of Haicom Electronics Corporation and the Razor IMU from Sparkfun Electronics. The HI-204III receiver can provide precise satellite positioning data with a continuous tracking of all satellites in view. The 20 parallel channels and 4000 search bins can provide rapid satellite signal acquisition within >40 seconds and >8 seconds in cold and hot start, respectively. With −159 dBm tracking sensitivity and 1 Hz update rate, it can provide good navigation in urban areas. The 9 DoF Razor IMU consists of ITG-3200 triple-axis gyro, ADXL345 triple-axis accelerometer, and a HMC5883L triple-axis magnetometer. It also has an ATmega328 on-board microprocessor with 13-bit resolution which can provide serial data output at maximum 57,600 bps.

The Razor IMU was programmed for AHRS following the procedure given in “Sensor Model and Attitude Heading Reference System” section as shown in Figure 1. The AHRS could provide pitch and roll and yaw information. Besides, it was also programmed to provide acceleration information in longitudinal axis. The GPS and the AHRS were integrated following the procedure given in [33] as shown in Figures 2 and 3. The IMU was set to capture data at 50 Hz. The AHRS and GPS assembly were rigidly fixed in the test vehicle. The test vehicle was driven in various roads of Selangor, Malaysia, that included road intersections, straight road, and curved roads.

**Figure 10:**
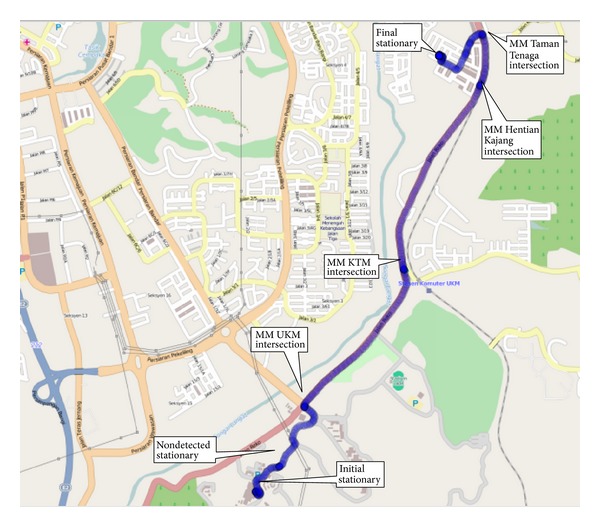
First test run in Jalan Reko, Kajang, Selangor, Malaysia, plotted in QGIS.

**Figure 11:**
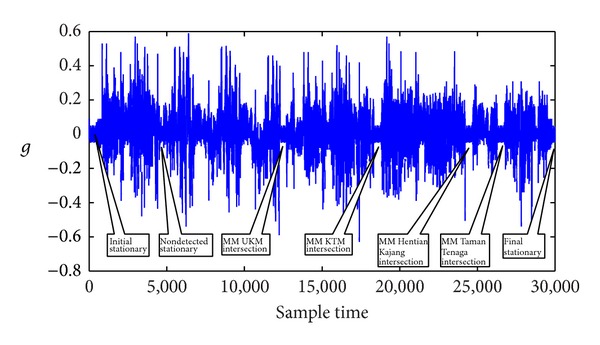
Stationary detection from acceleration signal in the first test.

**Figure 12:**
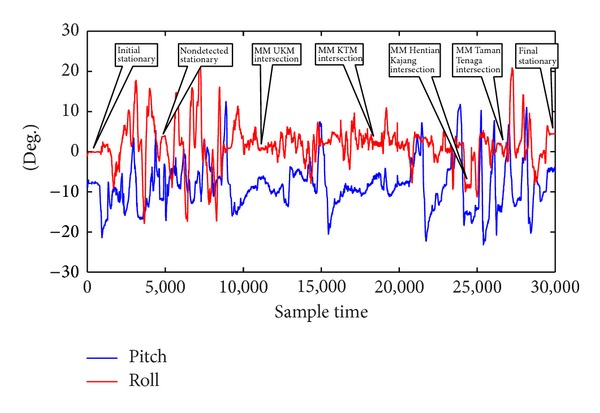
Stationary detection from pitch and roll signal in the first test.

**Figure 13:**
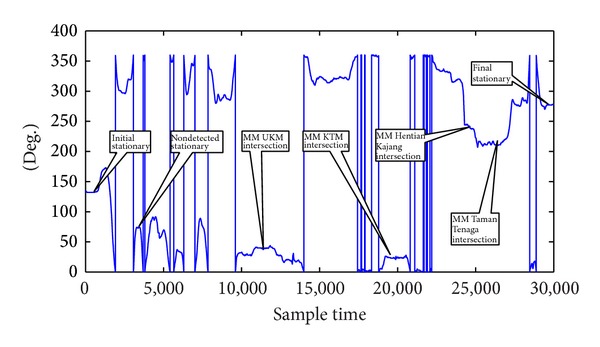
Stationary detection from heading signal in the first test.

The first test run was conducted in the Jalan Reko Road, Kajang, Selangor, Malaysia, as shown in Figure 10 plotted from the GPS positions in Quantum Geographical Information System (QGIS). The total duration of the test run was approximately 600 s. Seven stationary conditions were achieved during this time which included the initial stationary condition and the final stationary condition. The acceleration of the vehicle is shown in Figure 11, the roll and the pitch is shown in Figure 12, and the heading is shown in Figure 13. The acceleration, heading, and pitch and roll are shown in three different figures for the clarity of the changes. Besides the initial and final stop, the vehicle stopped in four road intersections. The vehicle also stopped once without the intersection.

The initial start-up place was not an intersection. But the vehicle remained in stationary position for more than 5 s. Thus, the proposed algorithm detected it as a stationary state from the acceleration, heading, and roll and pitch threshold. The vehicle again stopped without an intersection. But, this time, the vehicle stopped for less than 5 s. As there was no MM intersection and the duration was less than 5 s, the algorithm did not detect it as a stationary condition. Next the vehicle stopped at the intersection of UKM, UKM KTM station, and Hentian Kajang for more than 5 s. The MM algorithm identified these intersections correctly. The acceleration, heading, and pitch and roll also detected them as a stationary condition. Combining the MM intersection and the stationary condition from the threshold, the proposed algorithm detected the successful stationary condition. In the fourth intersection at Taman Tenaga intersection, the vehicle stopped only for 3 s. The MM algorithm could successfully detect the intersection. The threshold of the acceleration, heading, and pitch and roll also detected the stationary condition. The proposed algorithm does not confirm a stationary state only from the threshold if the stop condition is less than 5 s. As the MM algorithm also detected the intersection, the stationary algorithm confirmed the condition as stationary state combining with stationary state from the AHRS. But, in the second instance, the algorithm did not finalize the stationary state as the vehicle stopped for less than 5 s without any MM intersection. Finally, the vehicle stopped again without an intersection. But, during this time, the vehicle stopped for more than 5 s. Thus, the threshold of the acceleration, heading, and pitch and roll detected it as a stationary condition.

**Figure 14:**
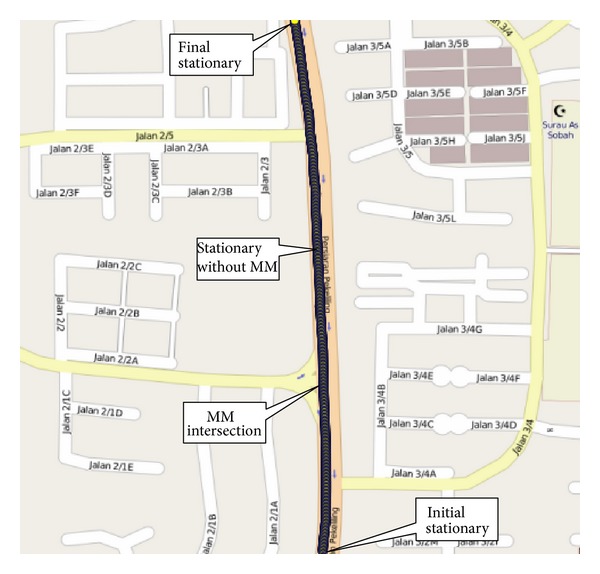
Second test run in Persiaran Pekeliling, Bangi, Selangor, Malaysia, plotted in QGIS.

**Figure 15:**
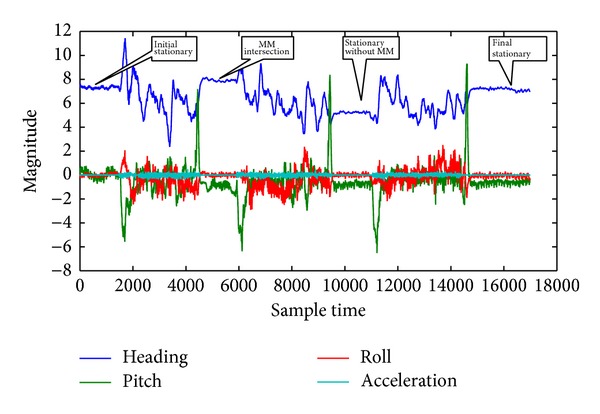
Stationary detection from acceleration, heading, and pitch and roll signal in the second test.

It is very difficult to visualize the stop condition from the heading of Figure 13 as the heading changes were from 0 to 360 degrees. As such, the second test was conducted in almost a northerly heading in the straight road of the Persiaran Pekeliling, Bangi, Selangor, as shown in Figure 14 in low speed so that the heading does not vary much. The duration of the second test drive was approximately 325 s. The test results including the acceleration, heading, and pitch and roll are shown in Figure 15. There was only one intersection on the tested road. However, the test vehicle intentionally stopped one more time. After starting the engine, the test vehicle was in stationary state for more than 20 s. The proposed algorithm detected it as a stationary state from the threshold of acceleration, heading, and pitch and roll as the vehicle stopped for more than 5 s even though the MM algorithm did not detect it as an intersection. After a while, the vehicle stopped in the first intersection around 4800 sample time. The stop condition was more than 5 s. The threshold of the heading change is apparent from Figure 15. The algorithm successfully detected it as stationary state as both the MM intersection and the threshold based stop detection coincided together. Next the vehicle stopped around 12800 sample time without an intersection for more than 5 s. Thus, the threshold algorithm correctly detected it as stationary state without the MM intersection. Finally, the vehicle again stopped without an intersection and the proposed algorithm detected it as a stationary state as shown in Figure 15.

**Figure 16:**
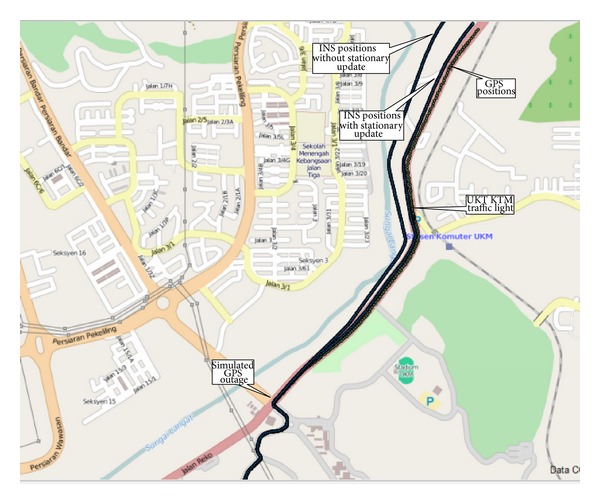
On vehicle performance test.

The effectiveness of stationary condition is noticeable during a sparse or unavailable GPS coverage. In urban areas, the absence of GPS aiding signal would drift the position. With stationary update, this drift can be reduced significantly. The navigational performance of the proposed stationary detection was evaluated through a test run. The across track errors were analysed as this was the only accuracy which could be evaluated through digital map reference of OpenStreetMap of QGIS. The test was carried out in the Jalan Reko Road, Kajang, Selangor, Malaysia, where the first stationary test was conducted as shown in Figure 15. Just from the first traffic signal of UKM, the GPS signal was obstructed intentionally to create the situation of a GPS outage condition. During this time, the navigational information was calculated only from the INS. From Figure 16, it is seen that the vehicle was drifting away gradually which reached 100 m after the intersection at UKM KTM station. Subsequently it drifted more than 200 m near Hentian Kajang without the stationary update solution. The vehicle was again driven on the same road starting from the same point. The GPS signal was also obstructed during the test run from the UKM traffic signal. But, this time, the navigational information was calculated from the INS using the stationary update. The algorithm detected the stationary state at the UKM traffic light. At the UKM KTM station intersection, the stationary state was again detected successfully. The error corrections were applied and, thus, the across track error was only 12 m. Before the Hentian Kajang traffic light, the across track error reached 20 m, whereas without the stationary update the across track error reached 200 m at the same point.

## 6. Discussions

It is evident from the experimental evaluation test run that the proposed algorithm successfully detected the stationary state and the IMU sensor errors were corrected accordingly without any false stationary detection. The stationary update decreased significant positioning errors by 180 m and thereby the navigational performance was increased by 90%. An effort is taken to compare these results with the previous reported research works. However, the majority of the previous research works are focused on the pedestrian and indoor navigation systems where the sensor errors are corrected when the foot of the pedestrian touches the ground. But the approaches for land vehicle systems are different. Thus, the proposed system is incomparable with the pedestrian and indoor navigation systems and is compared only with the land vehicle systems. However, the performance cannot be easily compared due to the lack of uniformity of the specifications, such as sensor price, sensor specification, number of sensors used, test vehicle, and route differences. Despite these constraints, the proposed system is compared with the other similar reported researches. In [16–19], controlled conditions are imposed to a stationary state by limiting the vibration and ensuring immobility. But these methods are not applicable in most of the land vehicle applications like intelligent transportation system, vehicle tracking systems, lane departure, and warning systems. Another method reported in [14] for mapping application also uses the forced stationary by alerting the user when the escalation of position error covariance crosses a certain threshold during a GPS outage [14]. Thus, the system in [14, 16–19] suffers from the disadvantages of not being the automated system as compared with the proposed automated stationary detection without any user intervention.

The GPS velocity threshold of more than 20 s is proposed in [20] where the GPS velocity is used to detect the stationary state. The stationary state is normally used to limit the divergence of the positional information during a GPS outage by resetting the IMU errors. Therefore, the approach in [20] is completely different than the proposed method and suffers from the disadvantage of not detecting the stationary state in a GPS outage condition. But the advantage of the proposed system is the stationary detection without a GPS signal and increasing the positional accuracy in a GPS outage condition by resetting the IMU errors during the stationary state. The GPS used in [20] was a high sensitivity GPS for the error correction of the gyroscopes and the across track error was 75 m. But, in the proposed method, the across track error is only 20 m.

In [21], the stationary state was detected only from the accelerometer data with a longer time window of 15 s. The disadvantage of such long time window is the chances of missing the stationary states as the vehicle may not be stationary for such a lengthy period. On the contrary, the advantage of the proposed system is the variable timing window of only 3 s for the combined AHRS and MM based technique and 5 s for only AHRS based technique. Moreover, the test run in [21] used a high sensitivity GPS and turn detection module with MM technique to reduce the mapping error and no complete GPS outage condition was evaluated. In a poor GPS condition, the across track reached 75 m in [21] which is much more compared with the 20 m across track of the proposed system. A pattern recognition approach from the accelerometers proposed in [22] only evaluates the false stationary detection rate which is 5%. On the contrary, the advantage of the proposed stationary detection is the elimination of the false stationary detection. The stationary states are detected from the variation of heading, and pitch and roll of an AHRS built from the IMU sensors. Besides, the MM algorithm detects the intersections where the vehicle is likely to stop. Combining these two results, the stationary state is detected with a smaller time window of 3 s. A longer timing window of 5 s is used when the stationary state is detected only from the AHRS. Thus, the false stationary detection is eliminated with the combination of two results and longer timing window only for AHRS based detection. The comparison of accuracy of the proposed method with other reported works is summarized in Table 3. Considering the above factors, the proposed system outperforms the previous known reported works.

## 7. Conclusions

Stationary updates augment the performance of INS during the nonavailability of aiding sensors. Automatic and reliable detection of stationary state is a challenging task as the elimination of false stationary detection is vital. The proposed acceleration and AHRS based stationary detection are a novel approach with the combination of MM intersection detection and variable timing window. Combination of MM intersection for intersection and the stationary detection from acceleration, heading, and pitch and roll eliminate the possibility of false stationary detection. The test results also demonstrated a successful detection of stationary states without any false stationary detection. The navigational performance of the proposed stationary detection was also evaluated through test run where the across track error was found to be drastically reduced by 90% with the stationary update. The comparison results also show that the proposed algorithm outperforms the previous known reported works. The proposed algorithm would help to reduce INS error and enhance the performance of the navigation system in GPS outage condition.

## Figures and Tables

**Table 1:** Matched sequence procedure (*N*).

*c*_1_^1^	*c*_1_^2^	*c*_1_^3^	*c*_2_^1^	*c*_2_^2^	*c*_3_^1^	*c*_3_^2^
0.8	0.5	0.6	0.3	0.2	0.1	0.7

**Table 2:** Initial observation (*V*, *F*_*t*_).

→*c*_2_^1^	→*c*_2_^1^	→*c*_2_^2^		→*c*_3_^1^	→*c*_1_^3^
*c*_1_^1^	0.5	0.8	*c*_2_^1^	0.2	0.2
*c*_1_^2^	0.3	0.1	*c*_2_^2^	0.3	0.5
*c*_1_^3^	0.4	0.8			

**Table 3:** Comparison of accuracy of the proposed method with other reported works.

Reference	Method	Across track error	False detection
[20]	GPS velocity >20 s	75 m	—
[21]	Accelerometer, 15 s timing window	75 m	—
[22]	Accelerometer	—	5%
This work	Acceleration, heading, roll, pitch and MM, variable time window (5 s and 3 s)	20 m	Nil

## References

[1] Brković M, Simić M (2014). Multidimensional optimization of signal space distance parameters in WLAN positioning. *The Scientific World Journal*.

[2] Dao T-K, Nguyen H-L, Pham T-T, Castelli E, Nguyen V-T, Nguyen D-V (2014). User localization in complex environments by multimodal combination of GPS, WiFi , RFID, and pedometer technologies. *The Scientific World Journal*.

[3] Pei F, Wu M, Zhang S (2014). Distributed SLAM using improved particle filter for mobile robot localization. *The Scientific World Journal*.

[4] Xia Y, Hu J, Fontaine MD (2013). A cyber-ITS framework for massive traffic data analysis using cyber infrastructure. *The Scientific World Journal*.

[5] Lin JY (2014). Using evolutionary computation on GPS position correction. *The Scientific World Journal*.

[6] Amin MS, Reaz MBI, Nasir SS (2014). Integrated vehicle accident detection and location system. *TELKOMNIKA Telecommunication, Computing, Electronics and Control*.

[7] Amin MS, Nasir SS, Reaz MMBI, Ali MAM, Chang T-G (2014). Preference and placement of vehicle crash sensors. *Technical Gazette*.

[8] Zhang Q, Niu X, Zhang H, Shi C (2013). Algorithm improvement of the low-end GNSS/INS systems for land vehicles navigation. *Mathematical Problems in Engineering*.

[9] Schwarz KP, El-Sheimy N Mobile mapping systems–state of the art and future trends.

[10] Fan Q, Li W, Hui J (2014). Integrated positioning for coal mining machinery in enclosed underground mine based on SINS/WSN. *The Scientific World Journal*.

[11] Gupta V (2009). *Vehicle Localization Using Low-Accuracy GPS, IMU and Map-Aided Vision*.

[12] Quinchia AG, Falco G, Falletti E, Dovis F, Ferrer C (2013). A comparison between different error modeling of MEMS applied to GPS/INS integrated systems. *Sensors*.

[13] Lan K-C, Shih W-Y (2013). On calibrating the sensor errors of a PDR-based indoor localization system. *Sensors*.

[14] Grejner-Brzezinska DA, Toth CK, Yi Y Bridging GPS gaps in urban canyons: can ZUPT really help?.

[15] Ramanandan A, Chen A, Farrell JA (2012). Inertial navigation aiding by stationary updates. *IEEE Transactions on Intelligent Transportation Systems*.

[16] Cheng J, de Wan FJ (1996). A fast initial alignment method for strapdown inertial navigation system on stationary base. *IEEE Transactions on Aerospace and Electronic Systems*.

[17] Li J, Tao R Initial alignment technology of strapdown inertial navigation system based-on stationary base.

[18] Nebot E, Durrant-Whyte H (1999). Initial calibration and alignment of low-cost inertial navigation units for land vehicle applications. *Journal of Robotic Systems*.

[19] Chuanbin Z, Weifeng T, Zhihua J (2004). A novel method improving the alignment accuracy of a strapdown inertial navigation system on a stationary base. *Measurement Science and Technology*.

[20] Mezentsev O, Lu Y, Lachapelle G, Klukas R Vehicular navigation in urban canyons using a high sensitivity GPS receiver augmented with a low cost rate gyro.

[21] Basnayake C, Mezentsev O, Lachapelle G, Cannon M (2005). An HSGPS, inertial and map-matching integrated portable vehicular navigation system for uninterrupted real-time vehicular navigation. *International Journal of Vehicle Information and Communication Systems*.

[22] Yu H (2008). *An algorithm to detect zero-velocity in automobiles using accelerometer signals [M.S. thesis]*.

[23] Chu H-J, Tsai G-J, Chiang K-W, Duong T-T (2013). GPS/MEMS INS data fusion and map matching in urban areas. *Sensors*.

[24] Grewal MS, Andrews AP, Bartone CG (2013). *Global Navigation Satellite Systems, Inertial Navigation, and Integration*.

[25] Lai Y-C, Jan S-S, Hsiao F-B (2010). Development of a low-cost attitude and heading reference system using a three-axis rotating platform. *Sensors*.

[26] Yoo TS, Hong SK, Yoon HM, Park S (2011). Gain-scheduled complementary filter design for a MEMS based attitude and heading reference system. *Sensors*.

[27] Farrell J (2008). *Aided Navigation: GPS with High Rate Sensors*.

[28] Park B, Lee J, Kim Y, Yun H, Kee C (2013). DGPS enhancement to GPS NMEA output data: DGPS by correction projection to position-domain. *Journal of Navigation*.

[29] Quddus MA, Ochieng WY, Noland RB (2007). Current map-matching algorithms for transport applications: state-of-the art and future research directions. *Transportation Research C: Emerging Technologies*.

[30] Sahawneh LR, Al-Jarrah MA, Assaleh K, Abdel-Hafez MF (2011). Real-time implementation of GPS aided low-cost strapdown inertial navigation system. *Journal of Intelligent and Robotic Systems: Theory and Applications*.

[31] Davidson P, Hautamäki J, Collin J, Takala J Improved vehicle positioning in urban environment through integration of gps and low-cost inertial sensors.

[32] Niu X, Nasser S, Gooddall C, El-Sheimy N (2007). A universal approach for processing any MEMS inertial sensor configuration for land-vehicle navigation. *Journal of Navigation*.

[33] Amin MS, Reaz MBI, Bhuiyan MAS, Nasir SS (2014). Kalman filtered GPS accelerometer based accident detection and location system: a low-cost approach. *Current Science*.

